# Friedreich ataxia in COVID-19 time: current impact and future possibilities

**DOI:** 10.1186/s40673-020-00127-9

**Published:** 2021-01-06

**Authors:** Tommaso Schirinzi, Andrea Sancesario, Enrico Castelli, Enrico Bertini, Gessica Vasco

**Affiliations:** 1 Department of Systems Medicine, University of Roma Tor Vergata, Via Montpellier, 00133 Rome, Italy; 2 Department of Neurosciences, IRCCS Bambino Gesù Children’s Hospital, Rome, Italy

**Keywords:** COVID-19, SARS-CoV-2, Friedreich ataxia, Ataxia, Physical activity, Technology

## Abstract

COVID-19 outbreak profoundly impacted on daily-life of patients with neurodegenerative diseases, including those with ataxia. Effects on interventional trials have been recently described. Conversely, changes in physical activity programs, which are crucial in care of ataxic patients, have not been assessed yet.

Here we used a structured electronic survey to interview twenty patients with Friedreich ataxia (FA) on changes in physical activity during the lockdown in Italy.

Regular physiotherapy was interrupted for most patients and up to 60% of them referred a substantial worsening of self-perceived global health. However, FA patients (especially those mildly affected) adopted voluntarily home-based training strategies and, in 30% of cases, used technology-based tools (TBTs) for exercise.

COVID-19 crisis thus disclosed the urgent need to support ataxic patients improving systems for remote physical activity and technology-based assistance.

## Main text

Maas and colleagues recently described the impact of COVID-19 on trials for patients with ataxia and indicated the remote assessment as the possible “exit-strategy” to continue experimentations [1]. Actually, COVID-19 lockdown had consequences also on other scheduled activities of ataxic patients, such as rehabilitation programs and physical training, which are core elements of both their care and health, especially in those suffering with Friedreich ataxia (FA) [2].

Since a specific assessment has not have been provided yet, here we aimed at evaluating the impact of pandemic on physical activity of FA patients. Therefore, we systematically interviewed by a structured survey twenty FA patients (female = 45%; age = 17 ± 7 years; disease duration = 7.8 ± 4.8 years; age at onset = 9.4 ± 4.5 years; last visit SARA score = 17.6 ± 6.9) from IRCCS Bambino Gesù Children’s Hospital (Rome, Italy). The questionnaire was sent by e-mail in the first week of June 2020 and returned in one week by all participants. We investigated the changes in physical activity during the lockdown in Italy (March – May 2020), the use of technology-based tools (TBTs) for home-based exercise, the self-perception of global health (as worsened or stable). The International Physical Activity Questionnaires – Short Form (IPAQ–SF) [3], which allows an objective measure of the individual amount of physical activity as Metabolic Equivalent (MET) min/week, was also included with reference to the entire lockdown period (as average of several weeks).

The study was conducted following local ethical standards and principles of Helsinki declaration. The informed consent was acquired for each participant.

Obtained variables were assessed by descriptive statistics, non-parametric and parametric tests, as appropriate. To identify predictors of physical activity amount, a linear regression model was set, using total MET as dependent variable and age, sex, disease duration and SARA score as independent variables. Group differences in physical activity amount were determined trough one-way ANCOVA, using total MET as independent variable and SARA score as main covariate. SPSS 23.0 was used for all analyses.

Because of the lockdown, the number of patients undergoing physiotherapy was reduced by 72.2% (from 18/20 to 5/20). Conversely, the number of those practicing physical exercise spontaneously increased from 12/20 to 15/20. Specifically, all active patients (*n* = 15) trained indoor: 78.6% did gymnastic exercises, 53.3% did walk, 35.7% used stationary-bike or treadmill, and 21% climbed the stairs.

The mean ± st.dev. METs level in all FA patients was 940.5 ± 873.4 min/week and none of age, sex, disease duration and SARA score predicted physical activity amount. However, active patients had SARA score (16.5 ± 6.8) lower, and METs level (1040 ± 843) higher than inactive patients (SARA: 24 ± 5.7, *p* < 0.05. METs: 420 ± 840, *p* = 0.05, adjusted for clinical severity).

**Fig. 1:**
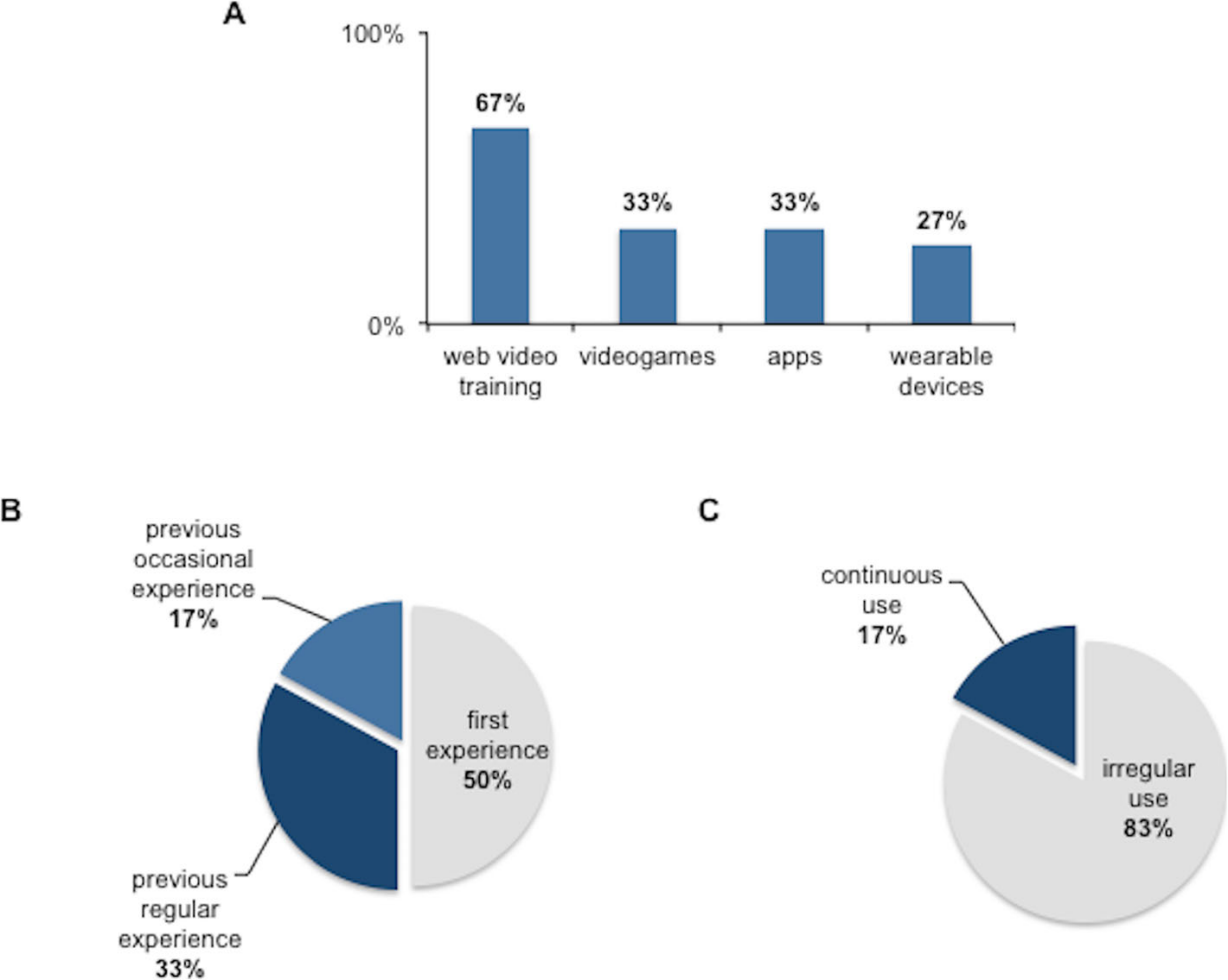
Use of TBTs. **a** Distribution of different TBTs’ use. TBTs might have been different among patients since they have been chosen autonomously and voluntarily, as available from web or other sources. **b** Experience with TBTs; 50% approached TBTs for the first time during the lockdown, others had previous experience (regular or occasional). **c** Regularity of TBTs use during the lockdown

BTs were used by 30% of patients, as represented in Fig. 1. Users had lower SARA score (12.3 ± 1.3; non-users = 20.9 ± 7.1, *p* = 0.01), while no differences emerged in METs between users and non-users (adjusted for clinical severity). All users had a positive final opinion on TBTs.

Up to 60% of patients reported self-perception of worsening in global health during COVID-19 lockdown but no predictors were identified.

Despite the small sample size, the absence of objective and comprehensive measures, and the selection bias due to age and modalities of participation (by e-mail), this survey showed how COVID-19 lockdown impacted on FA patients. Indeed a self-perception of worsening in the global wellbeing was complained by 60% of them.

Definitely many factors might have contributed, including psychological vulnerability and the altered structure of life around them; however, as it occurred in other conditions [4–6], the weight of regular physiotherapy and physical activities’ interruption can not be underestimated. In fact, physical training is critical for global health of FA patients [2], conditioning positively either personal abilities or quality of life [7–9].

This interview also disclosed a sort of resilient response from FA patients, especially from those with milder disease, which adopted different strategies for home-based training and approached TBTs for physical exercise.

COVID-19 crisis thus challenged the current care system of ataxic patients [10, 11] but, above all, opened the way to novel technologies in medical assistance. Consequently, there is an urgent need to develop systems for home-based physical training and improve those for remote assessment [12–16].

## Data Availability

Data are available on request.
